# A New Murine Model of Osteoblastic/Osteolytic Lesions from Human Androgen-Resistant Prostate Cancer

**DOI:** 10.1371/journal.pone.0075092

**Published:** 2013-09-19

**Authors:** Anaïs Fradet, Hélène Sorel, Baptiste Depalle, Claire Marie Serre, Delphine Farlay, Andrei Turtoi, Akeila Bellahcene, Hélène Follet, Vincent Castronovo, Philippe Clézardin, Edith Bonnelye

**Affiliations:** 1 Institut National de la Santé et de la Recherche Médicale (INSERM), Unité U1033, Lyon, France; 2 Université de Lyon, Lyon, France; 3 Université de Liège, Metastasis Research Laboratory, GIGA-CANCER, Liège, Belgium; University of Missouri-Columbia, United States of America

## Abstract

**Background:**

Up to 80% of patients dying from prostate carcinoma have developed bone metastases that are incurable. Castration is commonly used to treat prostate cancer. Although the disease initially responds to androgen blockade strategies, it often becomes castration-resistant (CRPC for Castration Resistant Prostate Cancer). Most of the murine models of mixed lesions derived from prostate cancer cells are androgen sensitive. Thus, we established a new model of CRPC (androgen receptor (AR) negative) that causes mixed lesions in bone.

**Methods:**

PC3 and its derived new cell clone PC3c cells were directly injected into the tibiae of SCID male mice. Tumor growth was analyzed by radiography and histology. Direct effects of conditioned medium of both cell lines were tested on osteoclasts, osteoblasts and osteocytes.

**Results:**

We found that PC3c cells induced mixed lesions 10 weeks after intratibial injection. *In* *vitro*, PC3c conditioned medium was able to stimulate tartrate resistant acid phosphatase (TRAP)-positive osteoclasts. Osteoprotegerin (OPG) and endothelin-1 (ET1) were highly expressed by PC3c while dikkopf-1 (DKK1) expression was decreased. Finally, PC3c highly expressed bone associated markers osteopontin (OPN), Runx2, alkaline phosphatase (ALP), bone sialoprotein (BSP) and produced mineralized matrix *in* *vitro* in osteogenic conditions.

**Conclusions:**

We have established a new CRPC cell line as a useful system for modeling human metastatic prostate cancer which presents the mixed phenotype of bone metastases that is commonly observed in prostate cancer patients with advanced disease. This model will help to understand androgen-independent mechanisms involved in the progression of prostate cancer in bone and provides a preclinical model for testing the effects of new treatments for bone metastases.

## Introduction

Bone is the most frequent site of prostate carcinoma metastases with bone metastases in up to 80% of advanced disease [1]. Surgical and hormonal therapies have shown beneficial effects only for early-stage hormone-responsive disease. Indeed, if the disease in most cases initially responds, it often progresses and become androgen independent. At that stage, patients with advanced disease often display osteoblastic or mixed lesions in bone [2,3]. The mechanisms by which prostate cancers are induced to metastasize to bone rely on a complex interplay between prostate cancer cells and the bone microenvironment [4]. Growth of prostate cancer cells alters bone remodeling (formation and resorption) by secreting factors that will directly affect osteoblasts (bone forming cells) and osteoclasts (bone resorbing cells). RANKL (Receptor activator of NF-kB ligand) stimulates osteoclasts differentiation and action while osteoprotegerin (OPG) acts as a decoy receptor for RANK (RANKL receptor). Therefore the balance between RANKL and OPG, that can be both produced by prostate cancer cells, is critical in controlling osteoclast activity and osteolysis in bone metastasis [4-6]. On the other side, pro-osteoblastic molecules can also be produced by prostate cancer cells. In fact, the first clinical studies to specifically target osteoblasts in patients with metastatic prostate cancer was based on endothelin-1 (ET1), a mitogenic factor for osteoblasts that can promote the growth of osteoblasts at metastatic sites [7,8]. In addition, transforming growth factor β (TGFβ), vascular endothelial growth factor (VEGF) are abundantly expressed by the prostate cancer cells and have a direct effect on osteoblast function [9,10]. The wingless (WNT) pathway that is implicated in osteoblastogenesis has been also implicated in the development of osteoblastic metastasis in prostate cancer [11]. Up-regulation of the WNT-family ligand WNT1 in prostate cancer cells and a decrease in the serum of the WNT antagonist dikkopf-1 (DKK1) expression has been reported in patients with advanced metastatic prostate carcinoma and is associated with osteoblastic lesions [12]. Finally prostate cancer cells that induce bone metastasis also express large amount of bone associated factors like osteopontin (OPN), osteocalcin (OCN) or bone sialoprotein (BSP) secreted in the bone matrix and that will contribute to promote their osteomimicry properties [13].

The majority of mixed bone metastases derived from prostate cancer mouse models are androgen sensitive and for that matter do not really mimic the clinical situation. We described the characterization of a new cell line (namely PC3c) that induce mixed skeletal lesions in animals that is derived from the human androgen independent AR-negative cell line PC3, known to induce pure osteolytic bone metastases.

## Materials and Methods

### Ethics statement

The mice used in our study were handled according to the rules of Décret N° 87-848 du 19/10/1987, Paris. The experimental protocol have been reviewed and approved by the Rhone-Alpes Regional Committee on the Ethic of Animal Experiments (Lyon, France) (Register Number: 0121). Animal experiments were routinely inspected by the attending veterinarian to ensure continued compliance with the proposed protocols. SCID mice, 6 weeks age, were housed under barrier conditions in laminar flow isolated hoods. Animals bearing tumor xenografts were carefully monitored for established signs of distress and discomfort and were humanely euthanized.

### Cell culture

PC3 cell line was obtained from the American Type Culture Collection (ATCC, Manassas, VA, USA). The PC3c cells, a subculture cell line of PC3 was isolated in our laboratory *in vitro* after single cell population culture. Consequently to spontaneous derivation of the cells, we finally obtained a subculture cell line named PC3c which was chosen based on its epithelial phenotype (Figure S1) [14,15]. The hormone dependent human prostate cancer VCAP cells were a generous gift of Pr M Cecchini (Department of Clinical Research, University of Bern, Bern, Switzerland) and was obtained from the American Type Culture Collection (ATCC, Manassas, VA, USA). VCAP were cultured in RPMI medium. PC3 and PC3c cells were routinely cultured in F12K nutrient mixture and DMEM medium (Life technologies, Carlsbad, CA, USA) respectively supplemented with 10% (v/v) fetal bovine serum (FBS; Perbio/Thermo scientific; Rockford, IL, USA) and 1% (v/v) penicillin/streptomycin (Life technologies, Carlsbad, CA, USA) at 37°C in a 5% CO2 incubator. PC3 and PC3c were also cultured upon osteogenic conditions for three weeks in the osteoblast medium supplemented with 50 µg/ml ascorbic acid (Sigma-Aldrich, Buchs, Switzerland). Ten mM sodium β-glycerophosphate (Sigma-Aldrich, Buchs, Switzerland) was added during 1 week at the end of the culture. PC3 and PC3c were continuously (day 1 to day 21) exposed to osteogenic conditions. For the visualization of mineralization, wells were fixed and stained with von Kossa and for ALP [16].

### Animal studies

For intra-osseous tumor xenograft experiments (Charles River Laboratories, Wilmington, MA, USA), a small hole was drilled with a 26-gauge sterile needle through the right tibia with the knee flexed in anesthetized 6- to 8-week-old SCID mice. Using a new sterile needle fitted to a 50-µl sterile Hamilton syringe (Hamilton Co.; Bonaduz, GR, Switzerland), a single-cell suspension (6x10^5^ in 15-µl PBS) of PC3 or PC3c cells was carefully injected in the bone marrow cavity. From week 2 after tumor cell inoculation, radiographs of anesthetized mice were weekly taken with the use of MIN-R2000 films (Kodak, Rochester, NY, USA) in an MX-20 cabinet X-ray system (Faxitron X-ray Corp, Tucson, AZ, USA). Animals were euthanized after 6 and 10 weeks for mice injected by PC3 and PC3c cells respectively. Microcomputed tomography analyses were carried out using a micro-CT scanner Skyscan 1174 (Skyscan; Kontich, Belgium). The X-ray tube was set to a voltage of 50 kV and a current of 800 µA. A 0.5 mm aluminum filter was used to reduce beam hardening artifacts. Samples were scanned in 70% ethanol with a voxel size of 20 µm. For each sample, 265 section images were reconstructed with NRecon software (version 1.6.1.8, Skyscan). Three-dimensional modeling and analysis of BV (Bone Volume)/TV (Total Volume) ratio (percentage of bone tissue) were obtained with the CTAn (version 1.9, Skyscan) and CTVol (version 2.0, Skyscan) software. The dissected bones were then processed for histological and histomorphometric analysis.

Subcutaneous injections of PC3c cells (10^6^ in 100µl PBS) were also performed in 6- to 8-week-old SCID mice. Animals were euthanized after 12 weeks and tumors were fixed and embedded in paraffin.

### Bone histomorphometry and histology

Tibia from animals were fixed, decalcified with 15% EDTA/ 0,4% PFA and embedded in paraffin. Five µm sections were stained with Goldner’s Trichrome and proceeded for histomorphometric analyses to calculate the TB (Tumor Burden)/STV (Soft Tissue Volume) ratio (percentage of tumor tissue). The *in situ* detection of osteoclasts was carried out on metastatic bone tissue sections using the tartrate-resistant acid phosphatase (TRAP) activity kit assay (Sigma-Aldrich, Buchs, Switzerland).

### Osteoclastogenesis assay

Primary bone marrow cells were obtained after tibia and femur bone marrow flushing from 6-week-old OF1 male mice. Cells were then cultured for 7 days, in differentiation medium: α-MEM medium containing 10% fetal calf serum (Life technologies, Carlsbad, CA, USA), 20 ng/mL of M-CSF (R&D Systems, Minneapolis, MN, USA) and 200 ng/mL of soluble recombinant RANK-L in presence or absence of conditioned medium extracted from PC3 and PC3c (25µg of proteins for each conditions) [17]. Medium was, first, changed every two days then from day 4 every days. After 7 days, mature multinucleated osteoclasts (OCs) were obtained and stained for TRAP activity (Sigma-Aldrich, Buchs, Switzerland), following the manufacturer’s instructions. Multinucleated TRAP-positive cells containing three or more nuclei were counted as OCs.

### Osteoblastogenesis assay

Calvaria of 3-day-old OF-1 mice were dissected then cells were enzymatically isolated by sequential digestion with collagenase, as described previously [18,19]. Cells obtained from the last four of the five digestion steps (populations II-V) were plated onto 24-well plates at 2x10^4^ cells / well. After 24 hours incubation, the medium including α-MEM medium containing 10% fetal bovine serum (Life technologies, Carlsbad, CA, USA) was changed and supplemented with 50µg/ml ascorbic acid (Sigma-Aldrich, Buchs, Switzerland) and with or without conditioned medium (25µg of proteins for each conditions) extracted from PC3 and PC3c. Medium was changed every two days for 15 days. 10mM sodium β-glycerophosphate (Sigma-Aldrich, Buchs, Switzerland) was added during 1 week at the end of the culture. At day 15, when bone mineralized nodules were formed, cells were then fixed and stained with von Kossa for quantification. ALP+ and bone mineralized nodules were then counted on a grid [16]. Results are plotted as the mean number of nodules ± SD of three wells for controls and each condition (PC3, PC3c) and were representative of two independent experiments. Osteocyte cell line MLO-Y4 were a generous gift of Pr L Bonewald (School of Dentistry, University of Missouri, Kansas City, MO, USA) and were cultured as described previously [20].

### Immunocytochemistry

PC3c tumors and metastatic tibia were fixed and embedded in paraffin. Five µm sections were subjected to immunohistochemistry using rabbit polyclonal antibodies anti human/ mouse osteopontin antibody (Bachem, Bubendorf, Switzerland), anti human Endothelin-1 antibody (Abbiotec, San Diego, CA, USA) and anti human OPG antibody (Abbiotec, San Diego, CA, USA). BSP antibody was a generous gift of Dr L Malaval (University of J Monnet, St Etienne, France). Sections were deparaffinized in methylcyclohexan, hydrated then treated with a peroxidase blocking reagent (Dako, Glostrup, Denmark). Sections were incubated with normal calf serum for 1 hour and incubated overnight at 4°C with primary antibodies (dilution: 1/100). Sections were incubated with secondary antibody HRP-conjugated donkey anti rabbit (Amersham/GE Healthcare; Chalfont St Giles, UK) (dilution 1/300) for 1 hour. After washing, the sections were revealed by 3,3’-diaminobenzidine (Dako, Glostrup, Denmark). Counterstaining was performed using Mayer’s hematoxylin (Merck, Whitehouse Station, NJ, USA).

### Real time RT-PCR

Total RNA was extracted with Trizol reagent (Life Technologies, Carlsbad, CA, USA) from PC3, PC3c, OBs, OCs and MLO-Y4 cells. Samples of total RNA (1 µg) were reverse-transcribed using random hexamer (Promega, Madison, WI, USA) and the first strand synthesis kit of Superscript^TM^ II (Life Technologies, Carlsbad, CA, USA). Real-time RT-PCR was performed on a Roche Lightcycler Module (Roche, Penzberg, Germany) with primers specific for human and mouse (see Tables S1 and S2). Real-time RT-PCR was carried out by using SYBR Green (Qiagen, Hilden, Germany) according to the manufacturer’s instructions with an initial step for 10 min at 95°C followed by 40 cycles of 20 sec at 95°C, 10 sec at Tm (see Tables S1 and S2) and 10 sec at 72°C. We verified that a single peak was obtained for each product using the Lightcycler Roche software. Amplimers were all normalized to corresponding L32 values. Data analysis was carried out using the comparative CT method: in real-time each replicate average genes CT was normalized to the average CT of L32 by subtracting the average CT of L32 from each replicate to give the ∆CT. Results are expressed as Log^-2 __CT^ with ∆∆CT equivalent to the ∆CT of the genes in PC3, PC3c or treated OBs, OCs and MLO-Y4 cells subtracting to the ∆CT of the endogenous control (non-treated OBs, OCs and MLO-Y4 cells respectively).

### Electron microscopy

PC3c cells were cultured on glass coverslips, then fixed for 1h in 2% glutaraldehyde in 0.1M of sodium cacodylate buffer at pH7.4. After three rinses in 0.2M saccharose in 0.1M of sodium cacodylate buffer, the cells were postfixed in 1% osmium tetroxyde in 0.15M cacodylate buffer, dehydrated in graded ethanol, then embedded in Epon. Ultrathin sections were counterstained with uranyl acetate and lead citrate, the examined under a 1200 EX JEOL electron microscope (Jeol, Tokyo, Japan).

### Fourier Transform InfraRed Microspectroscopy (FTIRM)

Undecalcified sections (2µm-thick) of tibia embedded in MMA were cut longitudinally with a microtome Polycut (Reichert-Jung, Leica, Germany), and stored between 2 glass slides. FTIRM was performed with a PerkinElmer GXII Auto-image Microscope (Norwalk, CT, USA), equipped with a cooled liquid nitrogen wide band Mercury Cadmium Telluride detector (7800-400 cm^-1^). Infrared measurements were performed on bone matrix (in cortical bone) around the tumor and on the tumor itself. Infrared measurement of cortical bone from sham mice was also collected. IR spectra were collected in transmission mode, at 4 cm^-1^ of spatial resolution, and 40 µm X 40 µm of spatial resolution. Contribution of air and MMA were subtracted from the original spectrum. Automatic baseline correction was performed on each IR spectrum with Spectrum software (PerkinElmer, Inc).

### Statistical analysis

Data were expressed as mean +/- SD, and analyzed statistically by one way analysis of variance (ANOVA) followed by post hoc t-tests or student t-test to assess the differences between groups for *in vitro* and *in vivo* studies. Statistical significance was taken as p<0.05.

## Results

### Expression of pro-osteoblastic factors by PC3c cells

**Figure 1:**
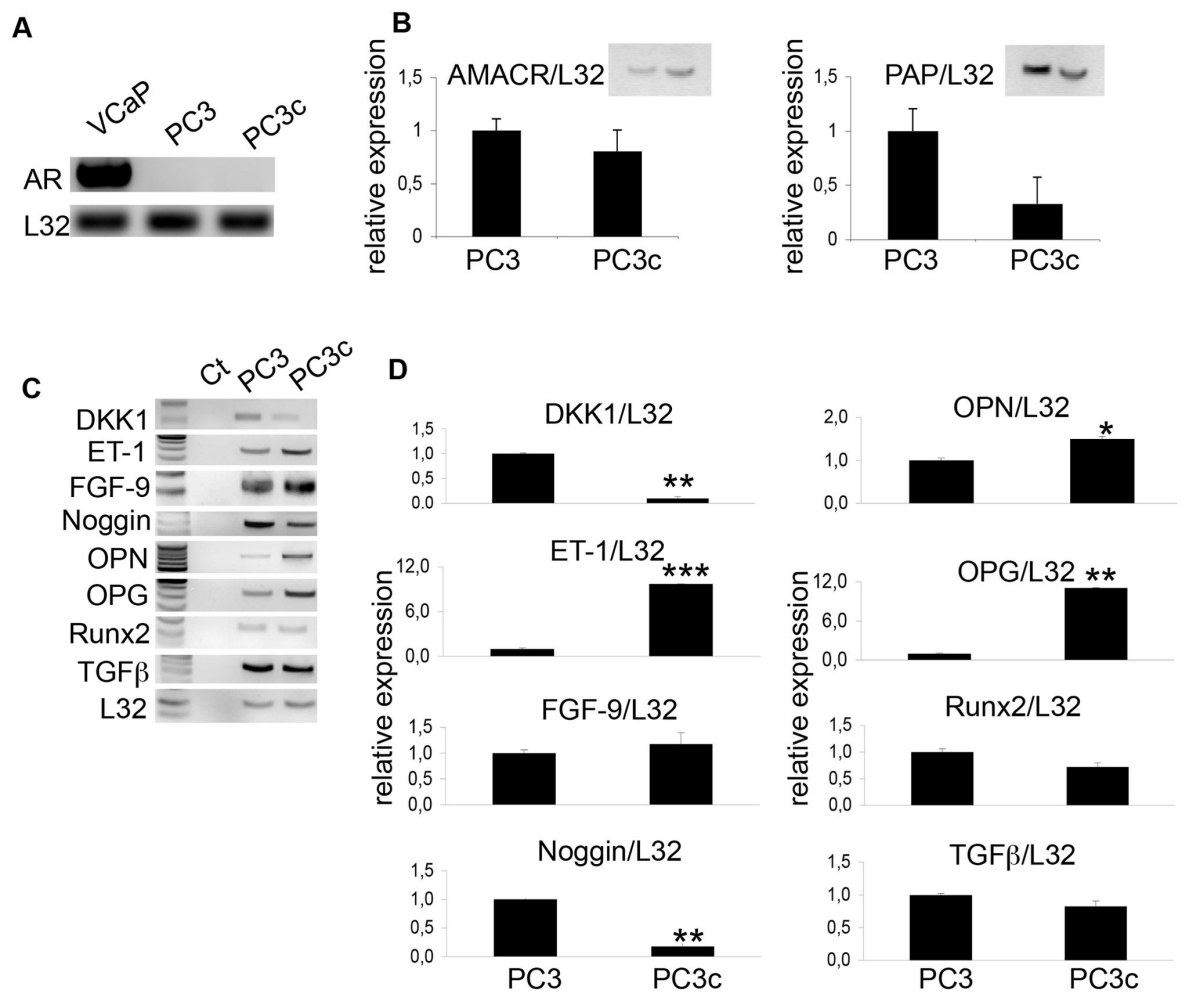
Expression of pro-osteoblastic factors by PC3c cells. Detection by real-time PCR of AR mRNA expression in PC3, PC3c and VCAP cancer cells lines (A), AMACR, PAP (B) and DKK1, ET-1, FGF9, Noggin, OPN, OPG, Runx2 and TGFβ mRNA expression (C and D) in PC3 and PC3c cancer cells lines. Genes expression was assessed by real-time PCR on triplicate samples and normalized against that of the ribosomal protein gene L32 *p<0.05; **p<0,001, ***p<0,0001.

From human androgen-resistant prostate cancer cell line PC3, we obtained after single cell population culture *in vitro* a new subculture cell line named PC3c cells that was chosen based on its epithelial phenotype (Figure S1). As expected and similarly to the parental PC3 cells, AR could not be detected by real-time PCR in PC3c while it was expressed in the hormone dependant cell line VCaP used as a positive control (Figure 1A). On the other hand, the prostate markers P504S (alpha methylacyl-coA racemase (AMACR)) and the prostatic acid phosphatase (PAP) were expressed in both cell lines confirming the prostate origin of the cells (Figure 1B) [21].

Characterization by real-time PCR of PC3c cells indicated that ET1 and OPG, two factors that have been implicated in the pathogenesis of osteosclerotic bone metastases from prostate cancer are overexpressed compared to the parental cell line PC3 (Figure 1C-D) while other factors such as fibroblast growth factor 9 (FGF9) and TGFβ are similarly expressed in both cell lines [8,22,23]. On the other hand, the expression of DKK1 and Noggin, two osteoblast inhibitors (respectively Wnts and bone morphogenetic protein (BMP) inhibitors), is decreased in PC3c versus PC3 (Figure 1 C-D) [24,25]. Moreover, PC3c similarly to PC3 cells expressed factors known to be implicated in prostate cancer osteomimicry such as OPN and Runx2. All together, these results suggest that PC3c cells may potentially induce osteoblastic lesions when compared with PC3 cells that are known to predominantly exhibit osteolytic lesions in bone.

### PC3c cells induce mixed osteoblastic/osteolytic bone lesions

**Figure 2:**
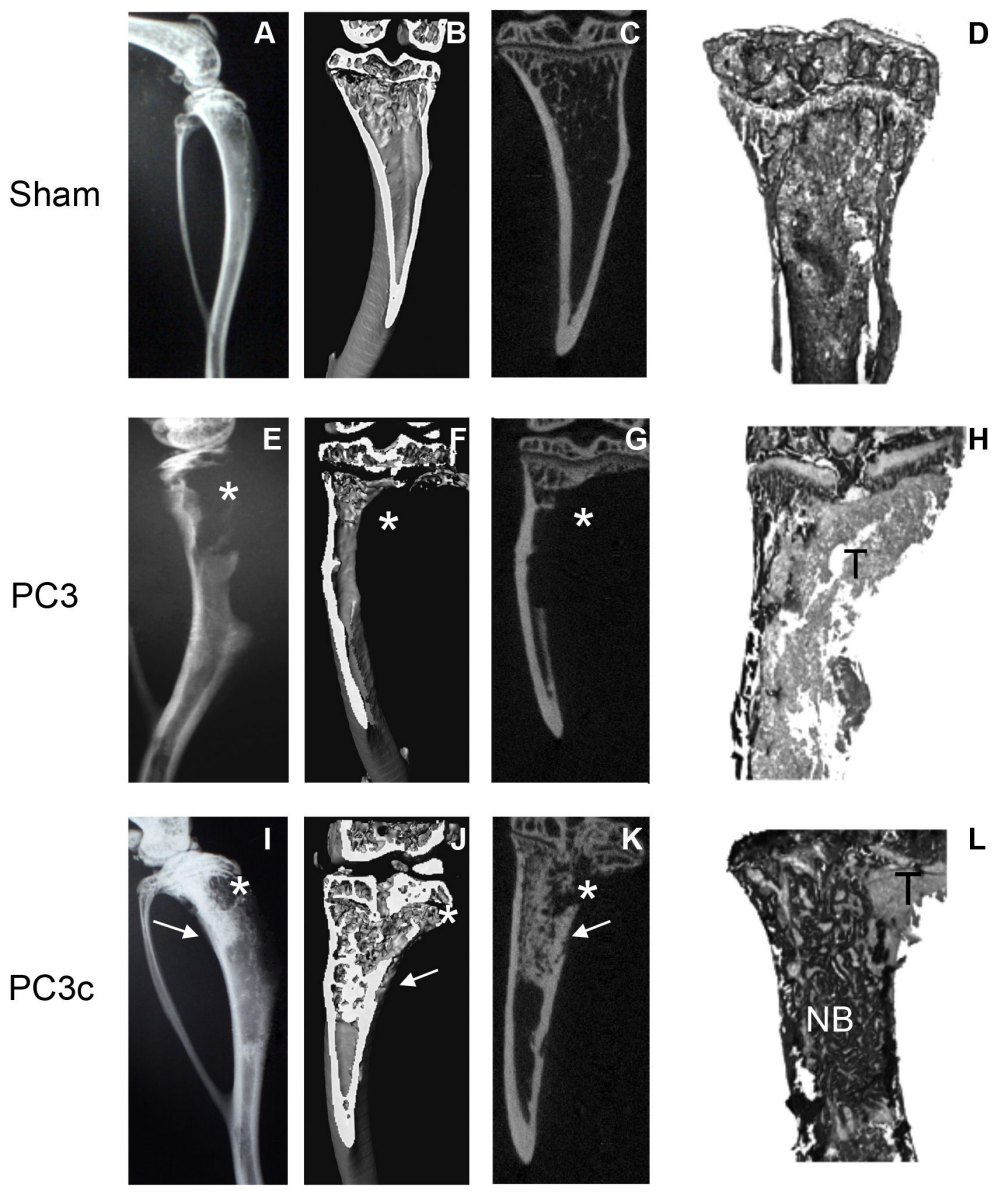
Induction of lytic and mixed bone lesions by PC3 and PC3c cells respectively after intratibial injection. (**A**) PC3 and PC3c cells were inoculated into male SCID mice; 10 weeks post inoculation, radiography revealed pure osteolytic lesions in mice injected with PC3 cells (n=6) (**E**) and mixed lesions in mice injected with PC3c cells (n=8) (**I**) compared to mice injected with PBS (n=10) (**A**) (see *(lysis) and white arrows (formation)). (**B,C- **F,G-J,K) Three-dimensional micro-CT reconstructions of tibiae and (**D**, **H**, **L**) histology after Goldner’s Trichrome staining confirmed the radiography results. T: Tumor; NB: New Bone.

In order to test the property of PC3c to induce bone lesions, intra-tibial injections were performed into male SCID mice. Ten weeks after tumor cell inoculation, radiographic analysis revealed that animals bearing PC3c tumors had bone lesions that included osteolytic and osteoblastic components (Figure 2I) while pure osteolytic lesions were observed in animal bearing PC3 tumors after 6 weeks (Figure 2E). The capacity of PC3 and PC3c to induce pure osteolytic and mixed lesions, respectively, was confirmed using 3D micro-CT reconstruction (Figure 2F-G and J-K) (bone volume, BV/TV, Table 1), histology (Figure 2H and L) and histomorphometric analyses of tibiae (skeletal tumor burden, TB/STV; Table 1). As expected no skeletal lesions were observed after PBS injection (Sham animals) (Figure 2A-D, Table 1). By immunohistochemistry, we confirmed, *in vivo*, that ET-1 and OPG were highly expressed in PC3c tumors (Figure S1, B, D) when compared with PC3 (Figure S2, A, C).

**Table 1:** Histomorphometric analysis of tibia with metastases induced by injection of PC3 and PC3c cells.

	BV/TV (%)	TB/STV (%)
Sham (n=10)	22,4 +/- 2,9	0
PC3 (n=6)	3,6 +/- 4,1***	72,6 +/- 6,6***
PC3c (n=8)	39,5 +/- 3,0*^$^	36,6 +/- 4,5***^$^

BV/TV: bone volume/ total volume. TB/STV: tumor burden/soft tissue volume. Sham were performed as control. *n* is the number of legs with bone metastases. *P<0,05; ***P<0,001 compared with Sham; ^$^P<0,001 compared with PC3.

### Bone remodeling stimulation by PC3c cells

**Figure 3:**
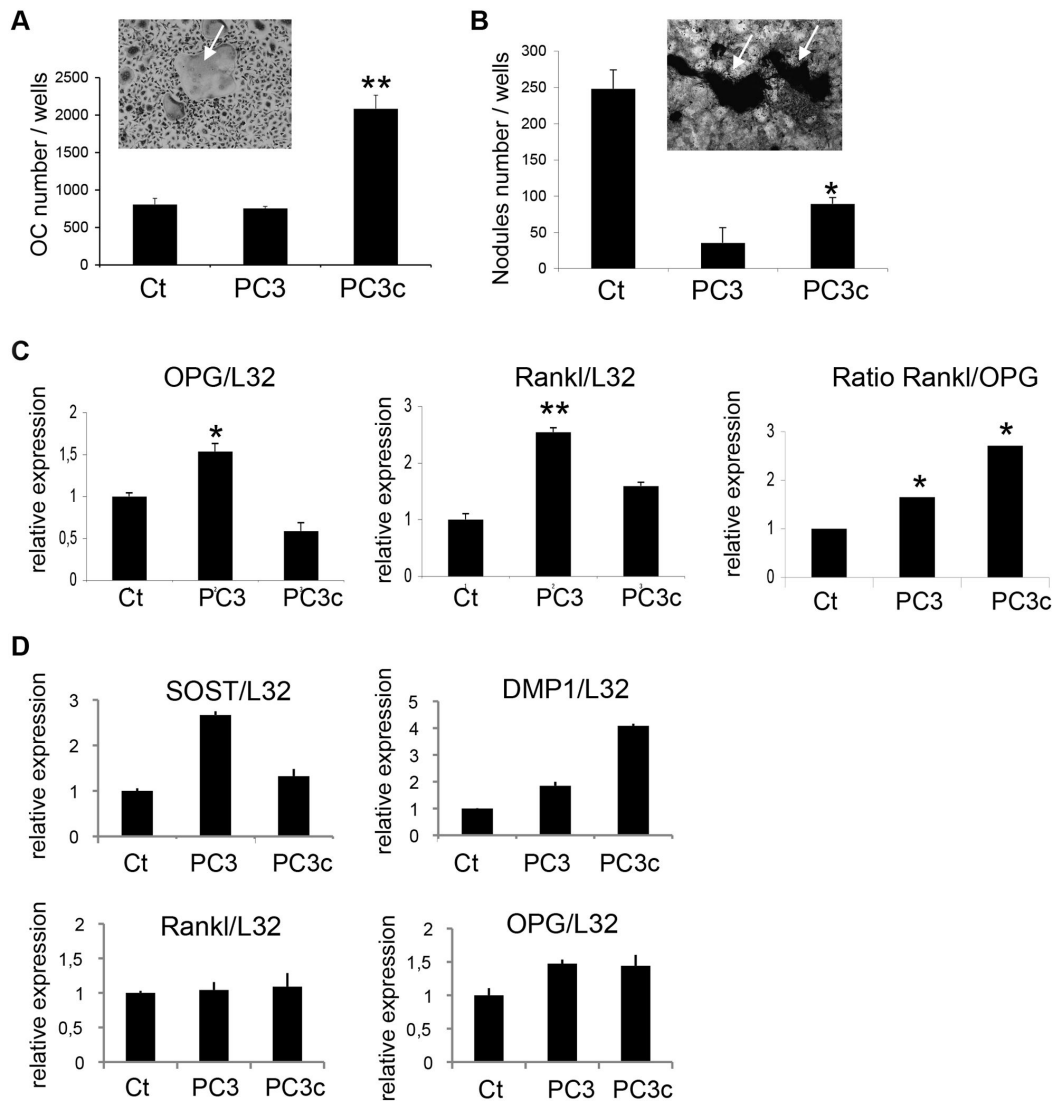
Stimulation of osteoclasts and osteoblasts by PC3c cells *in vitro*. (**A**) Primary mouse bone marrow cells were cultured in the presence of RANKL and M-CSF and treated or not (Ct) with conditioned medium obtained from PC3 and PC3c cells. More OCs (white arrow) were formed in cultures treated with PC3c conditioned medium compared to cultures treated with PC3 conditioned medium and Ct (ANOVA, p<0.0001). (**B**) Primary mouse calvaria cell cultures were treated from day 1-21 with conditioned medium obtained from PC3 and PC3c cell. Mineralized bone nodules were present and visualized by von Kossa staining at day 21 (see mineral in black, white arrows). Mineralized bone nodule formation was decreased when primary cells were treated with conditioned medium from any of the PC3/PC3c cells (compared with non-treated (Ct) cells); the decrease was less when PC3c cell conditioned medium was used (compared with PC3) (ANOVA, p<0.001 versus Ct and versus PC3). (**C**) PC3 conditioned media stimulated the expression of OPG and RANKL in primary OBs compared with non-treated (Ct) while PC3c conditioned media only inhibits the expression of OPG compared with Ct leading to an higher RANKL/OPG ratio in PC3c conditions. (**D**) Detection by real-time PCR of SOST, DMP1, OPG and RANKL mRNA expression in MLO-Y4 cells treated with PC3 and PC3c conditioned medium. Results are plotted as the mean number of OC ± SD and OB nodules ± SD of three wells for controls and each condition and are representative of two independent experiments. Genes expression was assessed by real-time PCR on triplicate samples and normalized against that of the ribosomal protein gene L32 *p<0.05; **p<0,001, ***p<0,0001.

**Figure 4:**
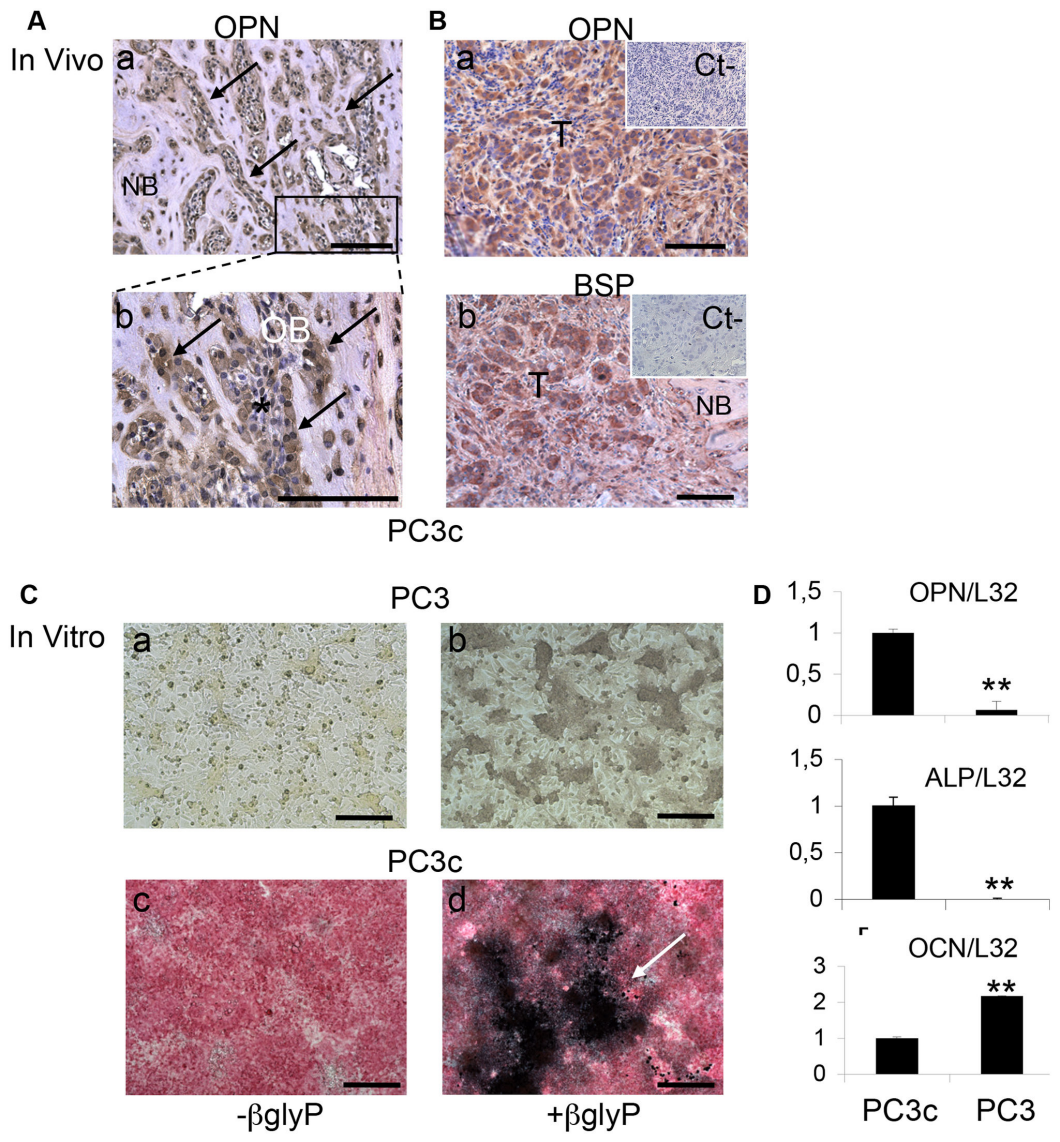
PC3c cells osteomimicry properties. (**A**) Immunodetection of OPN in OB in bone metastases induced by PC3c cells (see OBs **a** and **b** magnifications of **a**; see black arrows) (**A**) and in tumors cells (B a). Similarly to OPN, BSP expression is detected in tumors cells *in* *vivo* (**B**, **b**). (**C**) Similarly to primary mouse calvaria cells, PC3 and PC3c were cultured in osteogenic conditions for 21 days. ALP (a, c) and von Kossa staining (b, d) show high expression of ALP (c) and mineralization (white arrow) (d) in PC3c cells while no ALP expression (a) and mineralization were detected in PC3 cells (b). (**D**) Detection by real-time PCR of OPN, ALP and OCN mRNA expression in PC3c and PC3 cells cultured in osteogenic conditions for 21 days. Gene expression was assessed by real-time PCR on triplicate samples and normalized against that of the ribosomal protein gene L32 **p<0,001. Bar=200µm T: Tumor; OB: osteoblasts; NB: New Bone.

Given these data, we next asked whether PC3c could alter the bone resorbing cells, the osteoclasts (OCs) and the bone forming cells, the osteoblasts (OBs). Treatment of primary mouse bone marrow cells with RANKL, macrophage colony stimulating factor (M-CSF) and with the conditioned medium of PC3c stimulated more the formation of tartrate resistant acid phosphatase (TRAP)-positive multinucleated OCs compared with that observed with the conditioned medium of PC3 cells and untreated cells (Ct) (Figure 3A). On the other side, treatment of primary mouse calvaria cells cultured in osteogenic conditions with the conditioned medium of PC3c had less inhibitory effect on OB differentiation than conditioned medium of PC3 cells compared with untreated cells (Ct) (Figure 3B). Indeed, a high number of OBs was visualized using OPN immunostaining *in vivo* (Figure 4A a-b). Interestingly, PC3 conditioned medium stimulated OPG and RANKL expression by primary OBs while PC3c conditioned medium decreased OPG production leading to a stronger increase of RANKL/OPG ratio by OBs treated with PC3c conditioned medium compared with that of PC3 cells (Figure 3C). Consistent with these *in vitro* results, TRAP staining of tibial sections of metastatic legs from animals bearing PC3c showed high number of TRAP-positive multinucleated OCs compared with that observed in PC3 and Sham animals (Figure S3). Finally, semi-quantitative PCR performed on the osteocyte cell line, MLO-Y4, allowed us to show that sclerostin (SOST) and Dentin matrix acidic phosphoprotein 1 (DMP1) expression was stimulated after 24h of treatment with PC3 and PC3c conditioned medium respectively while OPG and RANKL expression was not affected (Figure 3D).

### PC3c cells induce robust osteoblastic reactions upon osteogenic conditions

**Figure 5:**
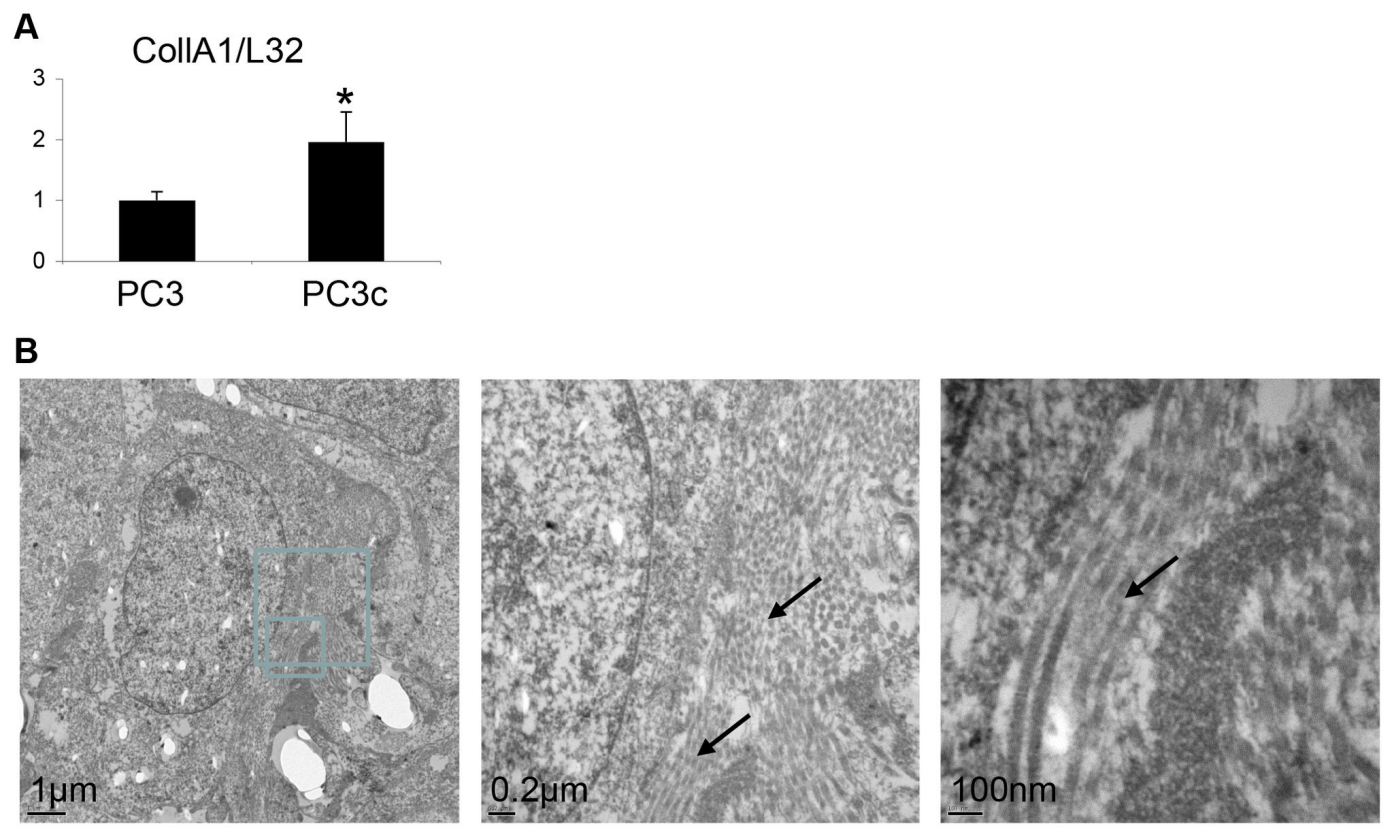
PC3c cells highly expressed type I collagen. (**A**) Detection by real-time PCR of type I collagen mRNA expression in PC3c and PC3 cells cultured in normal conditions. Gene expression was assessed by real-time PCR on triplicate samples and normalized against that of the ribosomal protein gene L32 *p<0,05. (**B**) Visualization of type I collagen in PC3c cells cultured on glass coverslip by electron microscopy (see black arrows).

Because PC3c cells induced new bone formation *in vivo*, we next tested whether they could produce OBs markers. After immunostaining of bone metastatic tissue sections, OPN and BSP were found expressed in PC3c cells *in situ* (Figure 4B a and b). Moreover after 3 weeks of culture, *in vitro*, upon osteogenic conditions including ascorbic acid and β-glycerophosphate (Fig 4C b and d), PC3c cells were revealed to be alkaline phosphatase (ALP)-positive (Fig 4Cc) and were able to form a calcified matrix positive for von Kossa staining (Fig 4C d), while PC3 were ALP-negative and did not induce matrix mineralization (Figure 4C a and b). Expression of ALP after ascorbic acid treatment was confirmed in PC3c cells by real-time PCR (Figure 4D). Similarly, OPN was highly expressed in PC3c compared to PC3 cells while OCN was expressed by both cells lines under these experimental conditions (Figure 4D), suggesting high osteomimicry property of PC3c compared to PC3 cells. Finally, Fourier Transform InfraRed Microspectroscopy (FTIRM) study on tumors obtained after subcutaneous injection of PC3c cells revealed the presence of amides I (mainly C=O stretching) and II (mainly N-H bending) and III (mainly C-N stretching and N-H bending) groups of proteins (Figure S4, see I and II red line) that usually correspond to the organic matrix (90% type I collagen) in bone (Figure S4, see Blue and black line). No phosphate or carbonate molecular vibrations were found, indicating the absence of mineral within the PC3c tumor *in vivo* (Figure S4). On the other side, new bone matrix obtained from mice tibia injected with PC3c cells showed the presence of mineral (Figure S4). Concomitantly to these result, high amount of Type I Collagen was found to be expressed by PC3c when compared with PC3 cells by real-time PCR *in vitro* (Figure 5A). Additionally, PC3c cells were shown surrounded by typical type I Collagen fibers *in situ* as judged by electron microscopy (Figure 5B see arrows and higher magnification). All together, these data suggest higher osteomimicry properties for PC3c compared with PC3 cells, thereby explaining at least in part, their capacity to induce mixed osteoblastic/osteolytic bone lesions.

## Discussion

In this study, we have established and characterized a new androgen-independent prostate cancer cell line (PC3c) which rapidly gives mixed bone lesions in male SCID mice, 10 weeks after intra-tibial tumor cells injection. This model may be useful to study cellular and molecular mechanisms that differ between androgen-dependent and androgen-independent prostate carcinomas when they metastasize to bone. In prostate cancer, androgen blockade strategies are usually used to treat osteoblastic bone metastases. However, responses to these therapies are often brief due to post-traductional modifications or mutations of AR that reduce ligand binding and inevitably lead to CRPC [26,27]. The role of AR pathway in the osteoblastic progression of prostate cancer is poorly understood because available models of mixed and pure osteoblastic lesions (C4-2B, PCa2B, VCaP) are mainly androgen responsive like [28,29]. Consequently, an active AR pathway is believed to be implicated in the osteoblastic progression of prostate cancer. However, concerning our PC3c model, this assumption does not occur as both cells lines PC3 and PC3c cell lines do not express AR. On the other side, AR negative models commonly used as PC3 and DU145 cells that derived from CRPC patients induced pure osteolytic lesions and do not reproduce what it is observed in clinic [30,31]. Then, in order to meet the need for clinically relevant models of prostate cancer-associated bone lesions, more recent hormone-independent models have been developed like MDA PCa 118 and Ace-1 that similarly to the PC3c model do not express AR and induce mixed lesions [22,32]. Nevertheless, osteogenesis that was induced by FGF-9 in the MDA PCa 118 model was not implicated in the osteoblastic response induced by PC3c as FGF-9 expression was not statistically significantly modulated between PC3 and PC3c cells.

Interestingly, when compared with PC3, PC3c cells highly expressed ET-1, a mitogenic factor for OB [33,34]. ET-1 is known to contribute to osteoblastic progression of prostate cancer cells by stimulating OB proliferation through the negative regulation of the inhibitor of the Wnt signaling, DKK1 which may explain, at least in part, the decrease of DKK1 observed in PC3c cells [35]. Moreover, a decrease of DKK1 serum level in patients with advanced prostate cancer has been reported to be associated with occurrence of osteoblastic lesions which suggests that DKK1 inhibition in PC3c cells may contribute to the osteoblastic phenotype induced by these cells [12]. BMPs have also been implicated in the formation of new bone induced by prostate cancer and the inhibition of BMPs by their inhibitor Noggin in C4-2B cells induces a decrease in the osteoblastic response, suggesting that the low expression level of Noggin in PC3c cells compared with PC3 cells may also contribute to the osteoblastic lesions induced by PC3c cells [4,10]. Finally the over-expression of OPG may also contribute to the osteoblastic response induced by PC3c cells by limiting OC differentiation. Based on OC and OB *in vitro* assay and TRAP staining *in vivo*, it appears that PC3c cells can also over stimulate osteoclastogenesis directly by acting on OC precursors and indirectly by increasing the RANKL/OPG ratio by OBs conducting to a general bone remodeling stimulation. On the other side, the RANKL/OPG ratio was not modulated by osteocytes like MLO-Y4 cells. Concerning osteocyte involvement into prostate cancer progression in bone, we found that the expression of SOST, an inhibitor of Wnt signaling, was induced by PC3 cells conditioned medium, suggesting a direct effect of osteocytes on OB differentiation during tumor bone progression. Meanwhile the expression of DMP1, a member of the small integrin-binding ligand N-linked glycoproteins (SIBLINGs) that is involved in phosphatemia regulation, was only stimulated by PC3c cells conditioned media which, combined with the expression of type I collagen by the tumor cells, may contribute to the formation of the new bone mineralized matrix observed *in vivo* and by infrared microspectroscopy [36,37].

Finally, PC3c cells had strong osteomimicry properties compared to PC3 cells, as judged by the expression of bone-associated markers such as OPN, BSP, Runx2, and type I collagen and by the ability of this cell line to form calcified matrix under osteogenic conditions, while PC3 are negative for both. Bone matrix proteins are also known to influence tumor localization in the skeleton and the phenotype of skeletal lesions. For example, OPN and BSP have been associated with breast and prostate cancer cells affinity for bone, migration and survival [38]. Moreover, differential expression of BSP and OPN has been shown to be implicated in the switch between osteolytic versus osteoblastic bone lesions. Indeed, strong BSP expression has been found mainly in prostate when compared with breast cancer lesions while high level of OPN was essentially shown in breast versus prostate cancer. Thus, associating BSP to sclerotic lesions as a stimulator of bone mineralization and OPN to osteolytic bone lesions as an activator of OC [39]. Differences into OPN expression level between PC3 and PC3c cells under normal conditions cannot explain the osteoblastic pattern observed in PC3c model and it is likely that OPN and BSP contribute to the osteomimicry properties of PC3c cells by promoting cell attachment and bone matrix mineralization, respectively. Additionally, type I collagen and ALP may also be part of this osteosclerotic pattern induced by PC3c cells by being involved in the new bone matrix formation and mineralization respectively.

In summary, we have established a new AR-negative prostate cancer cell line that is derived from PC3 cells and that recapitulates the osteoblastic phenotype of prostate cancer in bone. This new model may be helpful for the identification of new signaling pathways that are involved in the progression of prostate cancer in bone and may provide a valuable tool for investigating the mechanisms of androgen-independent prostate cancer cells osteomimicry in osteoblastic bone lesions. Finally, it can also provide a clinically relevant experimental model for testing the effects of new treatments for bone metastases.

## Supporting Information

Figure S1 **Epithelial phenotype of PC3c cells.** Detection by real-time PCR of E-Cadherin, N-Cadherin and vimentin mRNA expression in PC3 and PC3c cancer cells lines. Genes expression was assessed by real-time PCR on triplicate samples and normalized against that of the ribosomal protein gene L32 **p<0,001, ***p<0,0001.
(TIF)

Figure S2 **ET-1 and OPG expression by PC3c cells *in vivo*.** Immunostaining for ET-1 (**A**, **B**) and OPG (**C**, **D**) is higher in bone metastases induced by PC3c cells (B, D) compared to PC3 cells (A, C). Bar=200µm T: Tumor.
(TIF)

Figure S3 **Visualization of TRAP positive OC in bone metastases induced by PC3 and PC3c cells.** TRAP (red) staining of OCs (black arrow) realized in sections of tibiae taken from mice injected with Sham (A), PC3 (B) and PC3c cells (C). Bar=200µm T: Tumor.
(TIF)

Figure S4 **Identification of amide groups in PC3c subcutaneous tumors.** (**A**) IR spectra obtained on PC3c tumors (red curve), on cortical tibial bone matrix bone from Sham (Black curve) or PC3c mice (Blue curve) illustrates the presence of amide I and II and III groups usually corresponding to organic matrix (mainly to type I collagen) observed in bone matrix. As expected, mineral was shown by IR on bone matrix of tibia injected by PBS (Sham) (Black curve) or PC3c cells (Blue curve) (see peaks corresponding to ν_3_PO_4_, ν_2_CO_3_ and ν_4_PO_4_ groups) while it was not present in PC3c tumors (red curve; see ν_3_PO_4_, ν_2_CO_3_ and ν_4_PO_4_ groups). (**B**) Bone quality analysis performed by infrared microspectroscopy. No differences were observed between normal cortical bone and the new bone (NB) induced by PC3c tumor cells (n=2).
(TIF)

Table S1 **Mouse primers and using conditions.**
(DOC)

Table S2 **Human primers and using conditions.**
(DOC)

## References

[1] Vela I, Gregory L, Gardiner EM, Clements JA, Nicol DL (2007) Bone and prostate cancer cell interactions in metastatic prostate cancer. BJU Int 99: 735-742. doi:10.1111/j.1464-410X.2006.06670.x. PubMed: 17166237. 17166237 10.1111/j.1464-410X.2006.06670.x

[2] Roudier MP, Morrissey C, True LD, Higano CS, Vessella RL et al. (2008) Histopathological assessment of prostate cancer bone osteoblastic metastases. J Urol 180: 1154-1160. doi:10.1016/j.juro.2008.04.140. PubMed: 18639279. 18639279 10.1016/j.juro.2008.04.140 PMC2992811

[3] Keller ET, Brown J (2004) Prostate cancer bone metastases promote both osteolytic and osteoblastic activity. J Cell Biochem 91: 718-729. doi:10.1002/jcb.10662. PubMed: 14991763. 14991763 10.1002/jcb.10662

[4] Weilbaecher KN, Guise TA, McCauley LK (2011) Cancer to bone: a fatal attraction. Nat Rev Cancer 11: 411-425. doi:10.1038/nrc3055. PubMed: 21593787. 21593787 10.1038/nrc3055 PMC3666847

[5] Theriault RL (2012) Biology of bone metastases. Cancer Control 19: 92-101. PubMed: 22487971. 22487971 10.1177/107327481201900203

[6] Coleman R, Gnant M, Morgan G, Clezardin P (2012) Effects of bone-targeted agents on cancer progression and mortality. J Natl Cancer Inst 104: 1059-1067. doi:10.1093/jnci/djs263. PubMed: 22752060. 22752060 10.1093/jnci/djs263

[7] Jimeno A (2004) Atrasentan: targeting the endothelin axis in prostate cancer. Expert Opin Investig Drugs 13: 1631-1640. doi:10.1517/13543784.13.12.1631. PubMed: 15566319. 10.1517/13543784.13.12.1631 15566319

[8] Nelson JB, Hedican SP, George DJ, Reddi AH, Piantadosi S et al. (1995) Identification of endothelin-1 in the pathophysiology of metastatic adenocarcinoma of the prostate. Nat Med 1: 944-949. doi:10.1038/nm0995-944. PubMed: 7585222. 7585222 10.1038/nm0995-944

[9] Dai J, Hall CL, Escara-Wilke J, Mizokami A, Keller JM et al. (2008) Prostate cancer induces bone metastasis through Wnt-induced bone morphogenetic protein-dependent and independent mechanisms. Cancer Res 68: 5785-5794. doi:10.1158/0008-5472.CAN-07-6541. PubMed: 18632632. 18632632 10.1158/0008-5472.CAN-07-6541 PMC4432935

[10] Logothetis CJ, Lin SH (2005) Osteoblasts in prostate cancer metastasis to bone. Nat Rev Cancer 5: 21-28. doi:10.1038/nri1529. PubMed: 15630412. 15630412 10.1038/nrc1528

[11] Hall JM, Korach KS (2003) Stromal cell-derived factor 1, a novel target of estrogen receptor action, mediates the mitogenic effects of estradiol in ovarian and breast cancer cells. Mol Endocrinol 17: 792-803. doi:10.1210/me.2002-0438. PubMed: 12586845. 12586845 10.1210/me.2002-0438

[12] Chen G, Shukeir N, Potti A, Sircar K, Aprikian A et al. (2004) Up-regulation of Wnt-1 and beta-catenin production in patients with advanced metastatic prostate carcinoma: potential pathogenetic and prognostic implications. Cancer 101: 1345-1356. doi:10.1002/cncr.20518. PubMed: 15316903. 15316903 10.1002/cncr.20518

[13] Huang WC, Xie Z, Konaka H, Sodek J, Zhau HE et al. (2005) Human osteocalcin and bone sialoprotein mediating osteomimicry of prostate cancer cells: role of cAMP-dependent protein kinase A signaling pathway. Cancer Res 65: 2303-2313. doi:10.1158/0008-5472.CAN-04-3448. PubMed: 15781644. 15781644 10.1158/0008-5472.CAN-04-3448

[14] Flajollet S, Tian T, Flourens A, Tomavo N, Villers A et al. (2011) Abnormal expression of the ERG transcription factor in prostate cancer cells activates osteopontin. Mol Cancer Res 9: 914-924. doi:10.1158/1541-7786.MCR-10-0537. PubMed: 21669963. 21669963 10.1158/1541-7786.MCR-10-0537

[15] Tian TV, Tomavo N, Huot L, Flourens A, Bonnelye E et al. (2013) Identification of novel TMPRSS2:ERG mechanisms in prostate cancer metastasis: involvement of MMP9 and PLXNA2. Oncogene [Epub ahead of print]. doi:10.1038/onc.2013.176. PubMed: 23708657. 10.1038/onc.2013.176 23708657

[16] Bonnelye E, Chabadel A, Saltel F, Jurdic P (2008) Dual effect of strontium ranelate: stimulation of osteoblast differentiation and inhibition of osteoclast formation and resorption in vitro. Bone 42: 129-138. doi:10.1016/j.bone.2007.08.043. PubMed: 17945546. 17945546 10.1016/j.bone.2007.08.043

[17] Fradet A, Sorel H, Bouazza L, Goehrig D, Dépalle B et al. (2011) Dual function of ERRα in breast cancer and bone metastasis formation: implication of VEGF and osteoprotegerin. Cancer Res 71: 5728-5738. doi:10.1158/0008-5472.CAN-11-1431. PubMed: 21734015. 21734015 10.1158/0008-5472.CAN-11-1431

[18] Bonnelye E, Merdad L, Kung V, Aubin JE (2001) The orphan nuclear estrogen receptor-related receptor alpha (ERRalpha) is expressed throughout osteoblast differentiation and regulates bone formation in vitro. J Cell Biol 153: 971-984. doi:10.1083/jcb.153.5.971. PubMed: 11381083. 11381083 10.1083/jcb.153.5.971 PMC2174324

[19] Bellows CG, Aubin JE, Heersche JN, Antosz ME (1986) Mineralized bone nodules formed in vitro from enzymatically released rat calvaria cell populations. Calcif Tissue Int 38: 143-154. doi:10.1007/BF02556874. PubMed: 3085892. 3085892 10.1007/BF02556874

[20] Kato Y, Windle JJ, Koop BA, Mundy GR, Bonewald LF (1997) Establishment of an osteocyte-like cell line, MLO-Y4. J Bone Miner Res 12: 2014-2023. PubMed: 9421234. 9421234 10.1359/jbmr.1997.12.12.2014

[21] Chauchereau A, Al Nakouzi N, Gaudin C, Le Moulec S, Compagno D et al. (2011) Stemness markers characterize IGR-CaP1, a new cell line derived from primary epithelial prostate cancer. Exp Cell Res 317: 262-275. doi:10.1016/j.yexcr.2010.10.012. PubMed: 20974126. 20974126 10.1016/j.yexcr.2010.10.012

[22] Li ZG, Mathew P, Yang J, Starbuck MW, Zurita AJ et al. (2008) Androgen receptor-negative human prostate cancer cells induce osteogenesis in mice through FGF9-mediated mechanisms. J Clin Invest 118: 2697-2710. PubMed: 18618013. 18618013 10.1172/JCI33093 PMC2447924

[23] Kamiya N, Suzuki H, Endo T, Takano M, Yano M et al. (2011) Significance of serum osteoprotegerin and receptor activator of nuclear factor κB ligand in Japanese prostate cancer patients with bone metastasis. Int J Clin Oncol 16: 366-372. doi:10.1007/s10147-011-0193-7. PubMed: 21327451. 21327451 10.1007/s10147-011-0193-7

[24] Secondini C, Wetterwald A, Schwaninger R, Thalmann GN, Cecchini MG et al. (2011) The role of the BMP signaling antagonist noggin in the development of prostate cancer osteolytic bone metastasis. PLOS ONE 6: e16078. doi:10.1371/journal.pone.0016078. PubMed: 21249149. 21249149 10.1371/journal.pone.0016078 PMC3020964

[25] Hall CL, Bafico A, Dai J, Aaronson SA, Keller ET (2005) Prostate cancer cells promote osteoblastic bone metastases through Wnts. Cancer Res 65: 7554-7560. PubMed: 16140917. 16140917 10.1158/0008-5472.CAN-05-1317

[26] Anbalagan M, Huderson B, Murphy L, Rowan BG (2012) Post-translational modifications of nuclear receptors and human disease. Nucl Recept Signal 10: e001 PubMed: 22438791. 22438791 10.1621/nrs.10001 PMC3309075

[27] Saraon P, Jarvi K, Diamandis EP (2011) Molecular alterations during progression of prostate cancer to androgen independence. Clin Chem 57: 1366-1375. doi:10.1373/clinchem.2011.165977. PubMed: 21956922. 21956922 10.1373/clinchem.2011.165977

[28] Navone NM, Olive M, Ozen M, Davis R, Troncoso P et al. (1997) Establishment of two human prostate cancer cell lines derived from a single bone metastasis. Clin Cancer Res 3: 2493-2500. PubMed: 9815652. 9815652

[29] Korenchuk S, Lehr JE, MClean L, Lee YG, Whitney S et al. (2001) VCaP, a cell-based model system of human prostate cancer. In Vivo 15: 163-168. PubMed: 11317522. 11317522

[30] Stone KR, Mickey DD, Wunderli H, Mickey GH, Paulson DF (1978) Isolation of a human prostate carcinoma cell line (DU 145). Int J Cancer 21: 274-281. doi:10.1002/ijc.2910210305. PubMed: 631930. 631930 10.1002/ijc.2910210305

[31] Kaighn ME, Narayan KS, Ohnuki Y, Lechner JF, Jones LW (1979) Establishment and characterization of a human prostatic carcinoma cell line (PC-3). Invest Urol 17: 16-23. PubMed: 447482. 447482

[32] LeRoy BE, Thudi NK, Nadella MV, Toribio RE, Tannehill-Gregg SH et al. (2006) New bone formation and osteolysis by a metastatic, highly invasive canine prostate carcinoma xenograft. Prostate 66: 1213-1222. doi:10.1002/pros.20408. PubMed: 16683269. 16683269 10.1002/pros.20408

[33] Bagnato A, Loizidou M, Pflug BR, Curwen J, Growcott J (2011) Role of the endothelin axis and its antagonists in the treatment of cancer. Br J Pharmacol 163: 220-233. doi:10.1111/j.1476-5381.2011.01217.x. PubMed: 21232046. 21232046 10.1111/j.1476-5381.2011.01217.x PMC3087127

[34] von Schroeder HP, Veillette CJ, Payandeh J, Qureshi A, Heersche JN (2003) Endothelin-1 promotes osteoprogenitor proliferation and differentiation in fetal rat calvarial cell cultures. Bone 33: 673-684. doi:10.1016/S8756-3282(03)00215-1. PubMed: 14555273. 14555273 10.1016/s8756-3282(03)00215-1

[35] Clines GA, Mohammad KS, Bao Y, Stephens OW, Suva LJ et al. (2007) Dickkopf homologue 1 mediates endothelin-1-stimulated new bone formation. Mol Endocrinol 21: 486-498. PubMed: 17068196. 17068196 10.1210/me.2006-0346 PMC2013302

[36] Feng JQ, Ward LM, Liu S, Lu Y, Xie Y et al. (2006) Loss of DMP1 causes rickets and osteomalacia and identifies a role for osteocytes in mineral metabolism. Nat Genet 38: 1310-1315. doi:10.1038/ng1905. PubMed: 17033621. 17033621 10.1038/ng1905 PMC1839871

[37] Harris SE, Gluhak-Heinrich J, Harris MA, Yang W, Bonewald LF et al. (2007) DMP1 and MEPE expression are elevated in osteocytes after mechanical loading in vivo: theoretical role in controlling mineral quality in the perilacunar matrix. J Musculoskelet Neuronal Interact 7: 313-315. PubMed: 18094489. 18094489 PMC3357082

[38] Bellahcène A, Castronovo V, Ogbureke KU, Fisher LW, Fedarko NS (2008) Small integrin-binding ligand N-linked glycoproteins (SIBLINGs): multifunctional proteins in cancer. Nat Rev Cancer 8: 212-226. doi:10.1038/nrc2345. PubMed: 18292776. 18292776 10.1038/nrc2345 PMC2484121

[39] Carlinfante G, Vassiliou D, Svensson O, Wendel M, Heinegård D et al. (2003) Differential expression of osteopontin and bone sialoprotein in bone metastasis of breast and prostate carcinoma. Clin Exp Metastasis 20: 437-444. doi:10.1023/A:1025419708343. PubMed: 14524533. 14524533 10.1023/a:1025419708343

