# Supplementary figures and images for: A New Murine Model of Osteoblastic/Osteolytic Lesions from Human Androgen-Resistant Prostate Cancer
Source: PLoS One. 2013 Sep 19;8(9):e75092. doi: 10.1371/journal.pone.0075092 (PMC3777927; doi:10.1371/journal.pone.0075092)

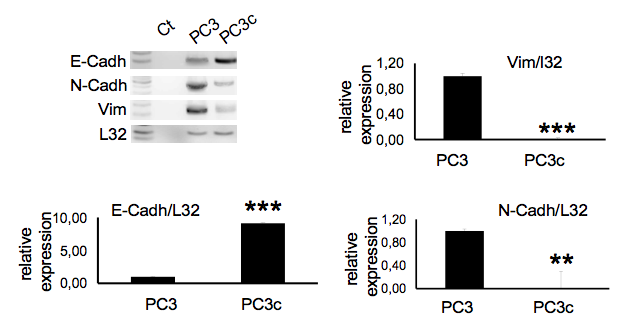

Supplement: Figure S1 — Epithelial phenotype of PC3c cells. Detection by real-time PCR of E-Cadherin, N-Cadherin and vimentin mRNA expression in PC3 and PC3c cancer cells lines. Genes expression was assessed by real-time PCR on triplicate samples and normalized against that of the ribosomal protein gene L32 **p<0,001, ***p<0,0001. (TIF) [file pone.0075092.s001.tif]

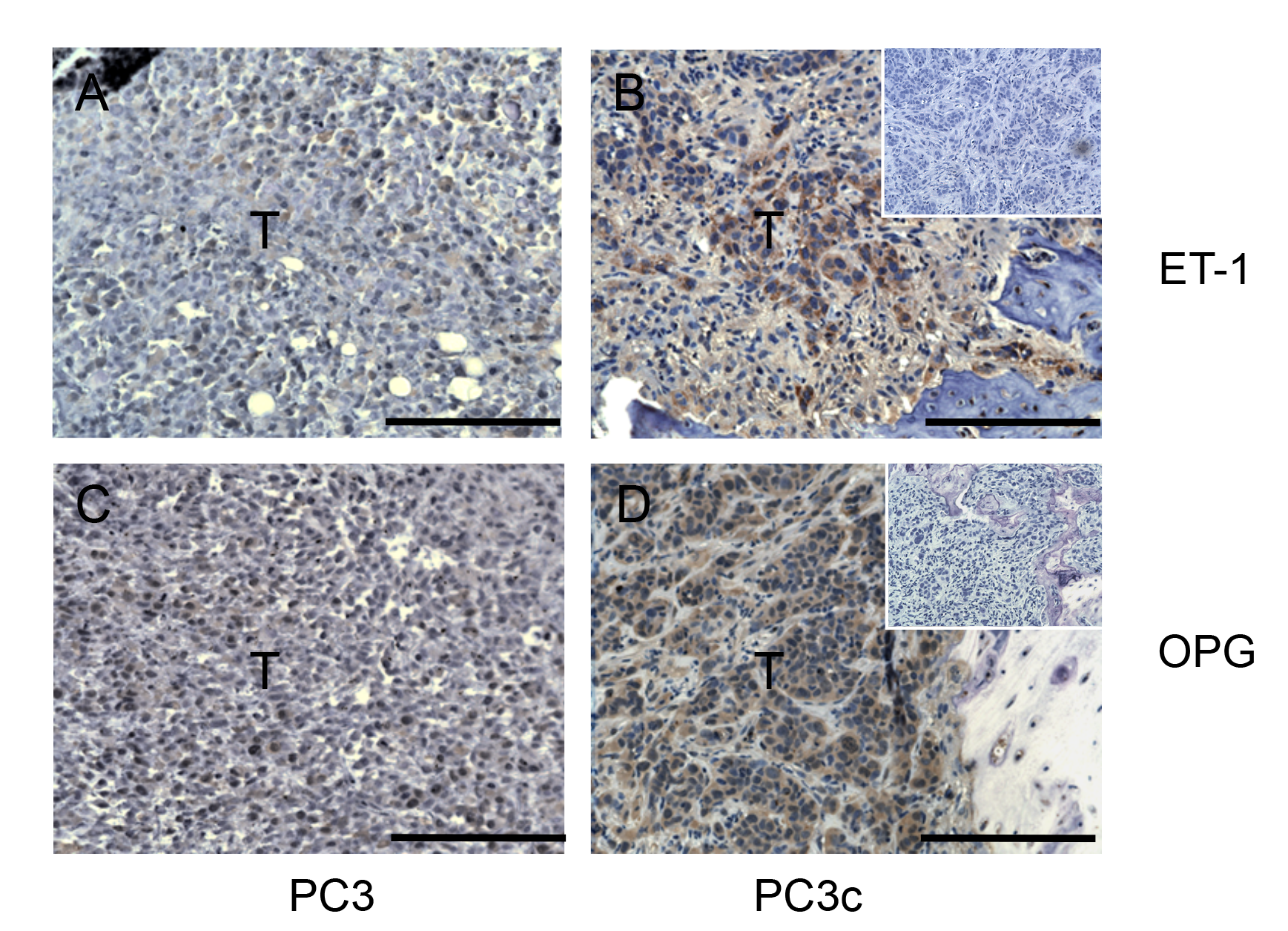

Supplement: Figure S2 — ET-1 and OPG expression by PC3c cells in vivo. Immunostaining for ET-1 (A, B) and OPG (C, D) is higher in bone metastases induced by PC3c cells (B, D) compared to PC3 cells (A, C). Bar=200µm T: Tumor. (TIF) [file pone.0075092.s002.tif]

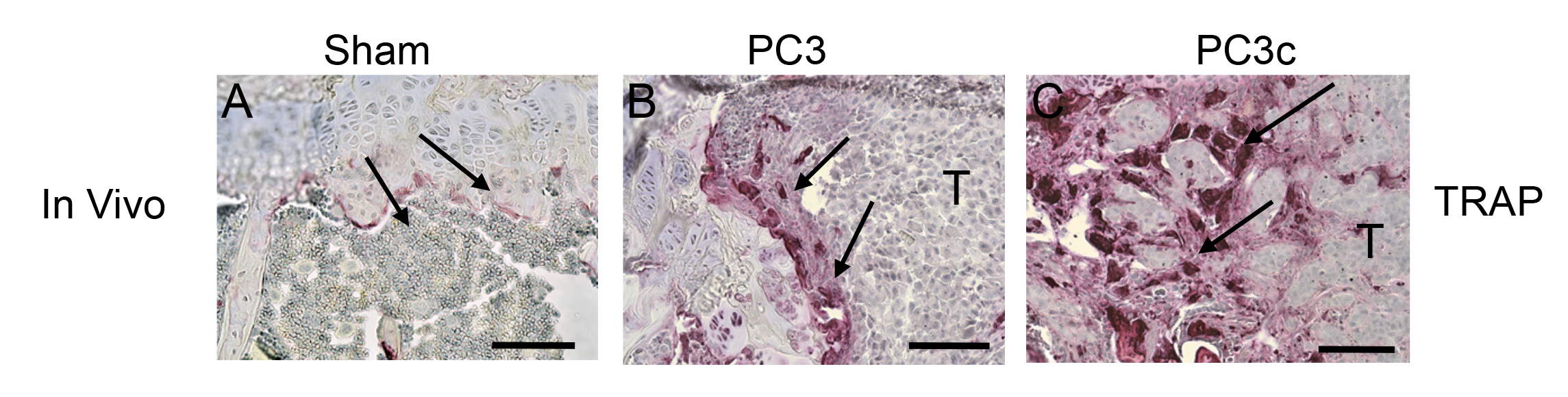

Supplement: Figure S3 — Visualization of TRAP positive OC in bone metastases induced by PC3 and PC3c cells. TRAP (red) staining of OCs (black arrow) realized in sections of tibiae taken from mice injected with Sham (A), PC3 (B) and PC3c cells (C). Bar=200µm T: Tumor. (TIF) [file pone.0075092.s003.tif]

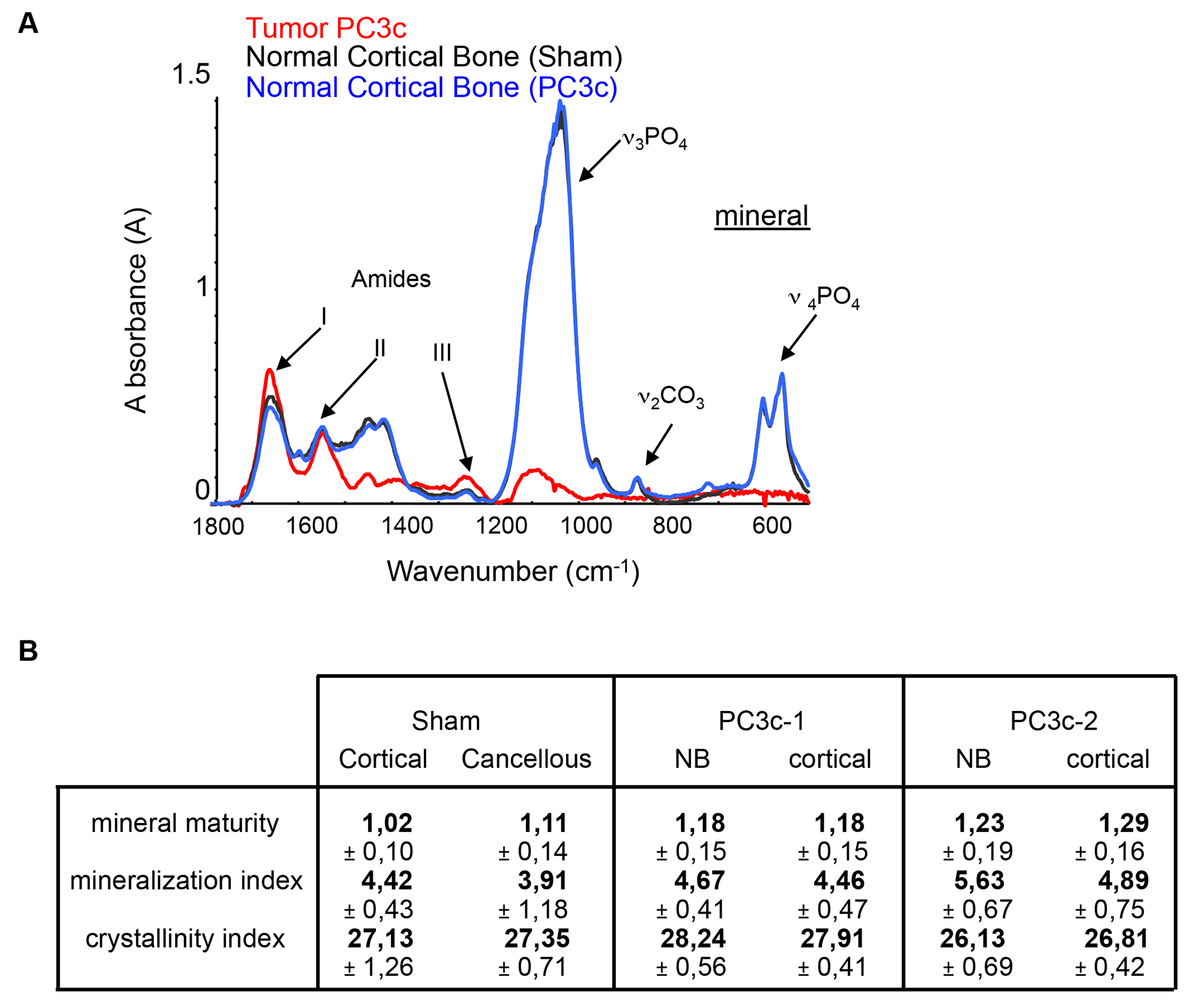

Supplement: Figure S4 — Identification of amide groups in PC3c subcutaneous tumors. (A) IR spectra obtained on PC3c tumors (red curve), on cortical tibial bone matrix bone from Sham (Black curve) or PC3c mice (Blue curve) illustrates the presence of amide I and II and III groups usually corresponding to organic matrix (mainly to type I collagen) observed in bone matrix. As expected, mineral was shown by IR on bone matrix of tibia injected by PBS (Sham) (Black curve) or PC3c cells (Blue curve) (see peaks corresponding to ν3PO4, ν2CO3 and ν4PO4 groups) while it was not present in PC3c tumors (red curve; see ν3PO4, ν2CO3 and ν4PO4 groups). (B) Bone quality analysis performed by infrared microspectroscopy. No differences were observed between normal cortical bone and the new bone (NB) induced by PC3c tumor cells (n=2). (TIF) [file pone.0075092.s004.tif]
