# Supplementary material for: A New Murine Model of Osteoblastic/Osteolytic Lesions from Human Androgen-Resistant Prostate Cancer
Source: PLoS One. 2013 Sep 19;8(9):e75092. doi: 10.1371/journal.pone.0075092 (PMC3777927; doi:10.1371/journal.pone.0075092)
Supplement: Table S1 — Mouse primers and using conditions. (DOC) [file pone.0075092.s005.doc]

| Gene | Primers | PCR cycles | T (°C) | Size (bp) | Reference |
| --- | --- | --- | --- | --- | --- |
| L32 | CAAGGAGCTGGAGGTGCTGC | 30 | 59 | 100 | NM_172086 |
|  | CTGCTCTTTCTACAATGGC |  |  |  |  |
| RANKL | GTGGTCTGCAGGATCGCTCTG | 40 | 63 | 286 | [1] |
|  | CGCTGGGCCACATCCAACC |  |  |  |  |
| OPG | TGTGTGACAAATGTGCTCC | 35 | 59 | 340 | [1] |
|  | GTCTCACCTGAGAAGAACCC |  |  |  |  |
| SOST | TTCAGGAATGATGCCACAGA | 35 | 54 | 179 | NM_024449.5 |
|  | GTCAGGAAGCGGGTGTAGTG |  |  |  |  |
| DMP1 | GTCACCACCACCACCCACGAAC | 35 | 64 | 149 | NM_016779.2 |
|  | AGAGTCCACCAGCCGGTCTGT |  |  |  |  |

1. Bonnelye E CA, Saltel F, Jurdic P. (2008) Dual effect of strontium ranelate: stimulation of osteoblast differentiation and inhibition of osteoclast formation and resorption in vitro. Bone 42: 129-138.
