# Supplementary material for: A New Murine Model of Osteoblastic/Osteolytic Lesions from Human Androgen-Resistant Prostate Cancer
Source: PLoS One. 2013 Sep 19;8(9):e75092. doi: 10.1371/journal.pone.0075092 (PMC3777927; doi:10.1371/journal.pone.0075092)
Supplement: Table S2 — Human primers and using conditions. (DOC) [file pone.0075092.s006.doc]

| Gene | Primers | PCR cycles | T (°C) | Size (bp) | Reference |
| --- | --- | --- | --- | --- | --- |
| L32 | CAAGGAGCTGGAAGTGCTGC | 25 | 62 | 100 | NM_000994.3 |
|  | CAGCTCTTTCCACGATGGC |  |  |  |  |
| AMACR | GCCCTTCCGCCGCGGTGTC | 30 | 61 | 180 | NM_000994.3 |
|  | CCAATTTTTGAGAGAACACCTG |  |  |  |  |
| OPN | CGCCGACCAAGGAAAACTCA | 38 | 64 | 604 | [1] |
|  | AACGGGGATGGCCTTGTATG |  |  |  |  |
| OPG | CACGACAACATATGTTCCGG | 40 | 61 | 320 | HSU94332 |
|  | TGTCCAATGTGCCGCTGCACGC |  |  |  |  |
| PAP | AAGGATTTGGCCAACTCACC | 30 | 61 | 210 | NM_001134194.1 |
|  | GGATAGGATTCCAGATGC |  |  |  |  |
| AR | CCTGATCTGTGGAGATGAAGGTTC | 40 | 56 | 400 | [1] |
|  | TGTCGTGTCCAGCACACACTACAC |  |  |  |  |
| ET-1 | TCCCACAAAGGCAACAGACCG | 40 | 59 | 217 | Y00749 |
|  | GCTGTTTCTCATGGTCTCCG |  |  |  |  |
| FGF9 | CAGTGTCCACGTGCTTATATAG | 40 | 60 | 210 | [2] |
|  | CACAGCCGATTTGGCATTCTG |  |  |  |  |
| Runx2 | GGAGTGGACGAGGCAAGAGTTT | 40 | 63 | 134 | NM_001024630.2 |
|  | AGCTTCTGTCTGTGCCTTCTGG |  |  |  |  |
| DKK1 | TAGCACCTTGGATGGGTATT | 25 | 58 | 143 | NM_012242 |
|  | ATCCTGAGGCACAGTCTGAT |  |  |  |  |
| TGFb1 | AGAAGCGGTACCTGAACCCG | 30 | 57 | 303 | NM_000660.4 |
|  | GCCGGTAGTGAACCCGTTG |  |  |  |  |
| Noggin | GAGCCGCCTCCGGAGAGAGACG | 40 | 60 | 261 | NM_005450 |
|  | TAGGGTCTGGGTGTTCGATG |  |  |  |  |
| ALP | AACACCAATGCCCAGGTCCC | 40 | 68 | 180 | NM_000478.3 |
|  | CACAGATTTCCCAGCGTCC |  |  |  |  |
| OCN | CCACCGAGACACCATGAGAGCCC | 40 | 64 | 200 | X53698.1 |
|  | GGGGACTGGGGCTCCCAGC |  |  |  |  |
| COLLA1 | TCGTGGAAATGATGGTGCTA | 40 | 59 | 349 | NM_000088.3 |
|  | ACCAGGTTCACCGCTGTTAC |  |  |  |  |
| E-Cadh | TGCCCAGAAAATGAAAAAGG | 35 | 60 | 180 | Z13009.1 |
|  | GTGTATGTGGCAATGCGTTC |  |  |  |  |
| N-Cadh | CACCCAACATGTTTACAATCAACAATGAGAC | 36 | 62 | 444 | M34064.1 |
|  | CTGCAGCAACAGTAAGGACAAACATCCTATT |  |  |  |  |
| Vimentin | GACAATGCGTCCTGTCCACAT | 35 | 55 | 230 | M14144.1 |
|  | TCTCCGCCTCCTGCAGGTTCTT |  |  |  |  |

1. Cheung CP, Yu S, Wong KB, Chan LW, Lai FM, Wang X, et al. Expression and functional study of estrogen receptor-related receptors in human prostatic cells and tissues. J Clin Endocrinol Metab. 2005 Mar;90(3):1830-44.

2. Li ZG, Mathew P, Yang J, Starbuck MW, Zurita AJ, Liu J, Sikes C, et al. Androgen receptor-negative human prostate cancer cells induce osteogenesis in mice through FGF9-mediated mechanisms. J Clin Invest. 2008 Aug;118(8):2697-710.
